# Super-resolution of X-ray CT images of rock samples by sparse representation: applications to the complex texture of serpentinite

**DOI:** 10.1038/s41598-023-33503-6

**Published:** 2023-04-24

**Authors:** Toshiaki Omori, Shoi Suzuki, Katsuyoshi Michibayashi, Atsushi Okamoto

**Affiliations:** 1 Department of Electrical and Electronic Engineering, Graduate School of Engineering, Kobe University, Kobe, 657-8501 Japan; 2 Center for Mathematical and Data Sciences, Kobe University, Kobe, 657-8501 Japan; 3 Center of Optical Scattering Image Science, Kobe University, Kobe, 657-8501 Japan; 4 Department of Earth and Planetary Sciences, Graduate School of Environmental Studies, Nagoya University, Nagoya, 464-8601 Japan; 5 Department of Environmental Studies for Advanced Society, Graduate School of Environmental Studies, Tohoku University, Sendai, 980-8579 Japan

**Keywords:** Solid Earth sciences, Scientific data

## Abstract

X-ray computed tomography (X-ray CT) has been widely used in the earth sciences, as it is non-destructive method for providing us the three-dimensional structures of rocks and sediments. Rock samples essentially possess various-scale structures, including millimeters to centimeter scales of layering and veins to micron-meter-scale mineral grains and porosities. As the limitations of the X-ray CT scanner, sample size and scanning time, it is not easy to extract information on multi-scale structures, even when hundreds meter scale core samples were obtained during drilling projects. As the first step to overcome such barriers on scale-resolution problems, we applied the super-resolution technique by sparse representation and dictionary-learning to X-ray CT images of rock core sample. By applications to serpentinized peridotite, which records the multi-stage water–rock interactions, we reveal that both grain-shapes, veins and background heterogeneities of high-resolution images can be reconstructed through super-resolution. We also show that the potential effectiveness of sparse super-resolution for feature extraction of complicated rock textures.

## Introduction

X-ray computed tomography (X-ray CT) is non-destructive method for providing us the three-dimensional structures. In decades, the applications of X-ray CT to geomaterials have been widely increasing, including rocks, sediments, and meteorites. In particular, the X-ray CT scanner is commonly applied to rock core samples by scientific drilling and/or developments of underground resources^1,2^. The downhole profiles of the X-ray CT values is used to extract the basic information on the physical properties of geological formations, and thus CT-value profiles lend themselves to geological interpretations^1,3–5^. For example, during the Oman drilling projects in 2016-2019, that drilled the crust and mantle sections of Oman ophiolite, the continuous X-ray CT images in total length exceeding 1000 m have been obtained by the onboard operation; the D/V *Chikyu* using a Discovery CT 750HD (GE Medical Systems)^2,6^. Crustal and mantle rocks taken from the Oman drilling projects showed various-scale structures that formed during igneous processes and water–rock interactions, including meter-scale igneous layering to millimeters scale veins to micro-scale mineral grain shapes, and nano-scale pores. However, as the voxel size of CT images at the D/V *Chikyu* is several hundreds micrometers, it is difficult to obtain the grain-scale information^2^. In contrast, more detailed CT images are obtained by the micro-CT at laboratories or synchrotron-based nano CT scanners at high energy acceleration institutes, although the size of analyzed samples is restricted. The hydrothermal experiments coupled with repeated CT imaging reveals the generations of porosity and evolution of fluid pathways during water–rock reactions^7,8^.

Traditionally, interpolation techniques are used to enhance the spatial resolution of images^9^, including bilinear interpolation and higher dimensional interpolation methods such as bicubic interpolation method. Bicubic interpolation uses a simple mathematical cubic function to interpolate data points on a regular two-dimensional grid. These interpolation methods can be applied to various images with low computational cost because they use common simple functions regardless of the target subject. Since these interpolation methods do not consider the characteristics of specific subjects, their simplicity limits their capability to enhance image quality, and they tend to generate excessively smooth images with artifacts^9^.

Super-resolution is a data-driven method based on machine learning for estimating a high-resolution image from a recorded low-resolution image^10,11^. In particular, learning-based super-resolution, which learns information about the subject in advance, is able to reconstruct images with higher accuracy by tailoring the learning process to the target image. Recently, super-resolution methods using deep learning methods have been proposed. Various types of deep learning methods have been applied to super-resolution, ranging from convolutional neural networks^12,13^ to generative adversarial networks^14^. However, the complexity of neural network architectures and the large numbers of network parameters mandate the need for a large amount of data to realize successful learning in most deep learning-based methods^15–18^. Sparse super-resolution is the another method that uses sparse coding to learn image characteristics for super-resolution. Sparse modeling is a framework that is suitable for applications with a sparsity of big data, where there is only a small number of explanatory variables^19^. It has been applied in many scientific fields including physics^20^, astronomy^21^, neuroscience^22–24^, and earth sciences^25^. In sparse super-resolution, an image is represented by the product of a dictionary obtained by learning and a coefficient vector so that the number of extracted basis images in the dictionary is minimized^10^. Thus, the image is represented under the constraint of a sparse coefficient vector.

In recent years, super-resolution methods have been developed for medical imaging techniques such as CT and magnetic resonance imaging. For the super-resolution of medical images, sparse modeling approaches have been developed, which is a white-box type (i.e., explainable) of machine learning^26^. For example, basis images, which explain the characteristics of target subject, are explicitly obtained and can be analyzed in sparse modeling approaches. In contrast, most deep learning approaches are the black-box type of machine learning. This means that for scientific purposes, the deep learning approaches have some disadvantages such as low explainability and interpretability owing to their complex mathematical frameworks^27–29^. For medical CT images, a previous study based on sparse modeling demonstrated that sharp and clear structures such as the sharp and clear boundaries between organs and clear structures in blood vessels are emphasized in the estimated super-resolution images as well as the super-resolution of natural standard images by assuming that only specific frequency elements are considered in the formulation of the super-resolution^26^. For CT images of rocks, however, it is important to estimate complex structures at high-resolution images such as rough, fine structures as well as macroscopic sharp boundaries such as the boundaries between open cracks and host rocks.

To overcome the scale-resolution problem for rock CT imaging, we propose a super-resolution technique using sparse representation and dictionary-learning. The proposed method can estimate the complex structure of rock rather than sharpening and smoothing the image like conventional interpolation methods. We demonstrated the effectiveness of the proposed method by applying it to the super-resolution of CT images of serpentinized dunite from the Oman ophiolite.

## Methods

### Serpentinized dunite of Oman ophiolite

In this study, we perform super-resolution of X-ray CT images for serpentinized dunite within the crust-mantle transition zone of the Oman ophiolite, which were suffered from the various stages of hydrothermal alteration. The Oman ophiolite is the best exposed section of oceanic lithosphere, and is located at the southearstern margin of Arabian Peninsula. The ophiolite is composed of pillow to massive submarine basalt, sheeted dike complex, cummulates and gabbros, and upper mantle rocks (dunite and harzburgite^30,31^). Hole CM1A was drilled at Wadi Zeeb, northern Sharqiyah ($22^{\circ }54.435^{\prime }\,\hbox {E}, 58^{\circ }20.149^{\prime }\,\hbox {N}$) in 2017 and the CM1A core was described aboard the D/V *Chikyu* in July to August in 2018. From 0 to 160 m depth, the core consists mainly of gabbroic rocks (olivine gabbro and troctolite), and from 160 to 310 m depth, the CM1A core consists mostly of dunite, which is classified as part of the crust- mantle transition zone. From 310 to 404 m depth, the core consists mainly of harzburgite, which is classified as part of the mantle sequence. Various stages of alteration reactions and veining that occurred over a range of temperatures and fluid infiltration conditions^32,33^.

### X-ray CT images

**Figure 1:**
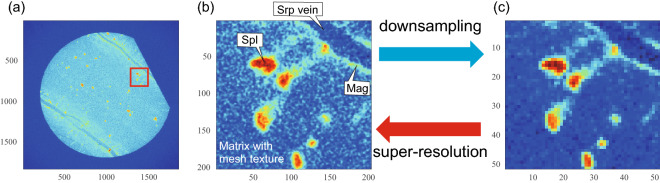
(**a**) X-ray CT images of the serpentinized dunite obtained by a medical CT scanner at *Chikyu*. The area of the patch area is indicated by the red square. (**b**) High resolution X-ray patch image of CM1A. *Srp* serpentine, *Spl* spinel, *Mag* magnetite. (**c**) Artificially created low-resolution X-ray patch image. Voxel size of the high-resolution image was 10 $\upmu \hbox {m}$.

We used the X-ray CT images of a serpentinized dunite sample (CM1A-90z02-48-53) taken from the crust-mantle transition zone at the drill site CM1A of the Oman Drilling Project^2,32,34^. The core sample of serpentinized dunite used in this study was scanned using a micro-focus X-ray CT scanner (Scan Xmate D225RSS270; Comscantecno) at Tohoku University (Fig. 1a, see Ref.^8^ for detailed information). The voltage was 120 kV, the current was 150 $\upmu \hbox {A}$, and the X-ray spot size was  9 $\upmu \hbox {m}$ (approximately half of 18 W). The pixel matrix was $1856 \times 1856$, and the voxel size was 10 $\upmu \hbox {m}$ (Fig. 1b). As the low-resolution images used in the super-resolution of this study, we also made the artificial degradation figures by averaging the sixteen pixels (Fig. 1c).

**Figure 2:**
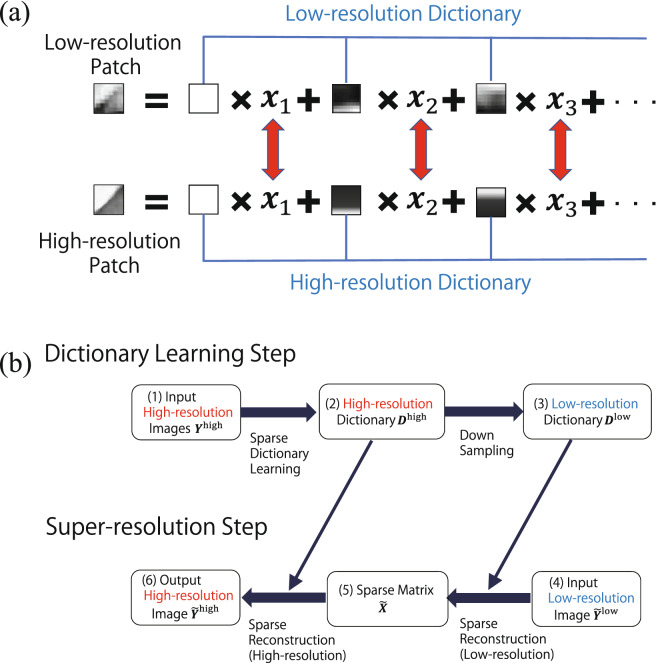
(**a**) Schematic illustration of the sparse super-resolution. (**b**) Flowchart of the proposed super-resolution algorithm for sparse representation.

**Figure 3:**
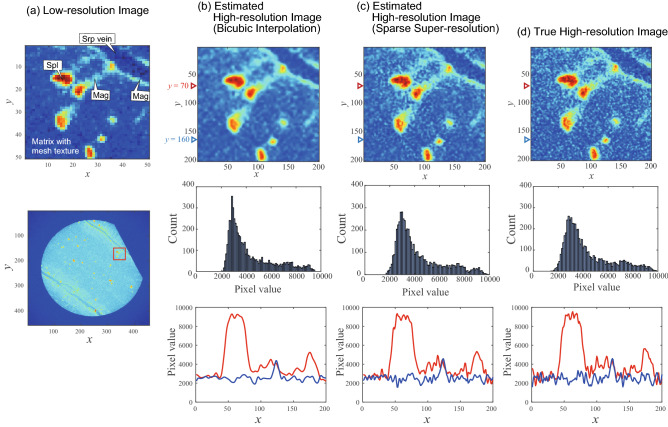
(**a**) Artificially created low-resolution X-ray image (top: enlarged image, bottom: entire image). *Srp* serpentine, *Spl* spinel, *Mag* magnetite. (**b**) Estimated high-resolution image by bicubic interpolation. (**c**) Estimated high-resolution image by sparse super-resolution. (**d**) True high-resolution image. For (**b**–**d**), top panel shows the high-resolution image, the middle panel shows a histogram of the pixel values in the two dimensional area of image), and the bottom panel shows the pixel values in the high-resolution image as a function of position *x* for a fixed value of *y* [$y=70$ (red line), and $y=160$ (blue line)]. Voxel size of the high-resolution image was 10 $\upmu \hbox {m}$.

**Figure 4:**
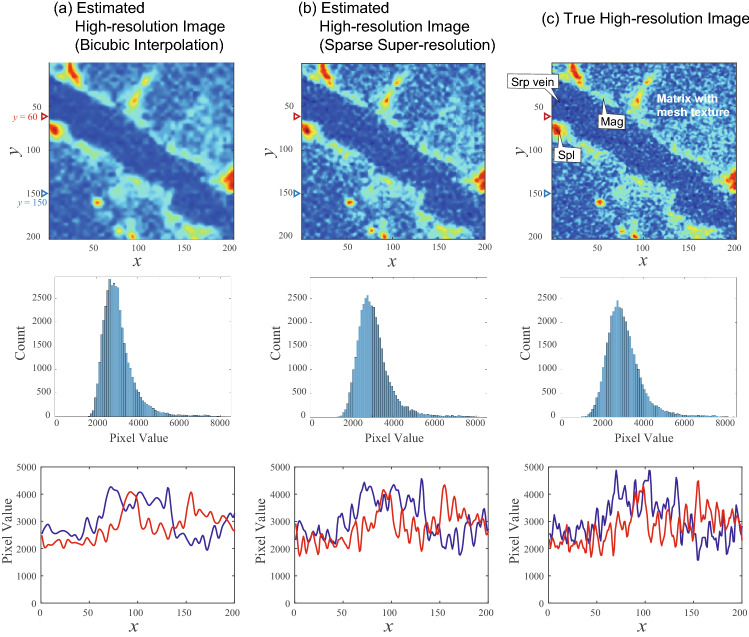
Top: high-resolution images estimated by existing and proposed methods. Fine structures seen in true image (**c**) is reconstructed by the proposed method (**b**), whereas a simplified image (**a**) is obtained by means of the existing method. Middle: histograms of pixels in the two dimensional area of image). Bottom: pixel values as a function of position *x* for a fixed value of *y* [$y=60$ (red line), and $y=150$ (blue line)]. *Srp* serpentine, *Spl* spinel, *Mag* magnetite. Voxel size of the high-resolution image was 10 $\upmu \hbox {m}$.

**Figure 5:**
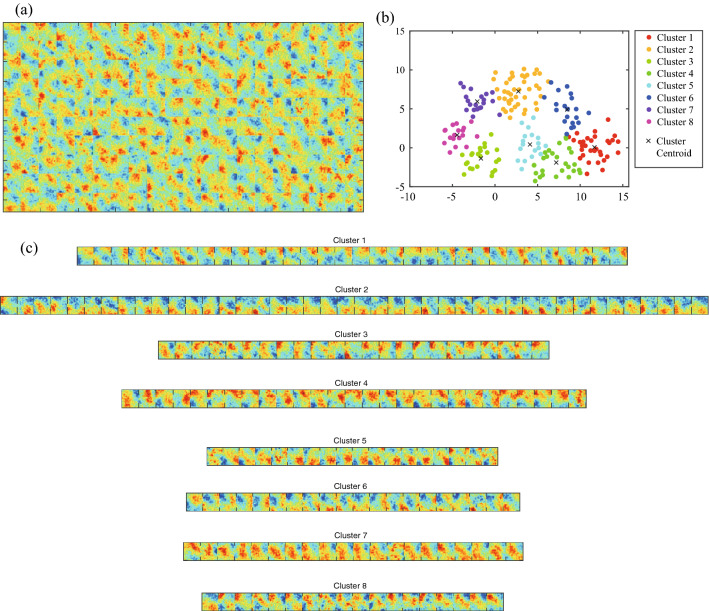
Dictionary trained by using rock CT images. (**a**) Basis images obtained by dictionary learning. (**b**,**c**) Clustering and visualization of basis images in the dictionary obtained from the rock CT images. In subfigures, (**b**) clustering results, and (**c**) basis images for each cluster are shown. In subfigure (**b**), each multi-dimensional vector for respective basis image is mapped into two-dimensional space by the dimension reduction method called *t*-SNE.

**Figure 6:**
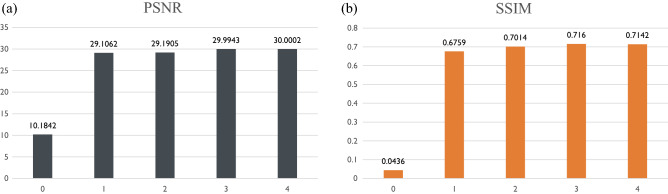
Quantitative evaluation indices for the discrepancy between true and estimated high-resolution CT images. (**a**) PSNR and (**b**) SSIM are shown as functions of the number of iterations.

The serpentinized dunite sample was completely serpentinized, and no relic of olivine or pyroxenes. The sample is mainly composed of serpentine minerals, brucite, magnetite and Cr-rich spinel^34^. The matrix parts of the sample show the mesh textures composed of $\hbox {lizardite}+\hbox {brucite}+\hbox {magnetite}$, showing the heterogeneities in scales of the original olivine grain or less. The matrix is cut by the later stage serpentine ($\hbox {antigorite}+\hbox {chrysotile}$) veins with thickness of 1 mm. Around this veins, bright brucite-rich reaction zone is developed with fine grained magnetite^34^. Cr-rich spinel is a subhedral grains with a size of 30 $\upmu \hbox {m}$. Around the serpentine veins, the rims of the spinel grained are replaced by magnetite and trails of magnetite formed within the blanches of the serpentine veins.

### Super-resolution method by sparse expression of rock CT images

Here, we describe the framework of sparse super-resolution for rock CT images. The detailed mathematical formulation is given in the Supplementary Material. In sparse image representation, natural images are expressed by a small number of basis images^19,23,35^. Each small area of a natural image called a patch, $\varvec{y}_i \in \{1,2,\ldots ,P \}$ (*P*: the total number of patches), which can be expressed by basis images $\left\{ \varvec{d}_1, \varvec{d}_2, \ldots ,\varvec{d}_D \right\}$ with a sparse vector (*D*: the total number of basis images):

1 $$\begin{aligned} \varvec{y}_i =x_{i,1} \varvec{d}_1 +x_{i,2} \varvec{d}_2 +\cdots +x_{i,D} \varvec{d}_D \end{aligned}$$

where $\varvec{x}_i =\left\{ x_{i,1},x_{i,2},\ldots ,x_{i,D} \right\}$ is a sparse vector in which most of the elements are zero. In both high-resolution and the corresponding low-resolution images, a sparse vector is assumed to be common as follows (Fig. 2a):

2 $$\begin{aligned} \varvec{y}_i^{\textrm{high}}= & {} x_{i,1} \varvec{d}_1^{\textrm{high}} +x_{i,2} \varvec{d}_2^{\textrm{high}} +\cdots +x_{i,D} \varvec{d}_D^{\textrm{high}} \end{aligned}$$

3 $$\begin{aligned} \varvec{y}_i^{\textrm{low}}= & {} x_{i,1} \varvec{d}_1^{\textrm{low}} +x_{i,2} \varvec{d}_2^{\textrm{low}} +\cdots +x_{i,D} \varvec{d}_D^{\textrm{low}} \end{aligned}$$

The super-resolution based on sparse representation comprises two steps (Fig. 2b): dictionary learning to obtain basis images by using high-resolution images, and super-resolution to reconstruct a high-resolution image by transferring sparse coefficients obtained from the low-resolution image representation image.

The dictionary learning step obtains a dictionary comprising the basis images $\varvec{D}^{\textrm{high}}=\left\{ \varvec{d}_{1}^{\textrm{high}}, \varvec{d}_{2}^{\textrm{high}},\ldots , \varvec{d}_{D}^{\textrm{high}} \right\}$ from a set of patch images $\varvec{Y}^{\textrm{high}}=\left\{ \varvec{y}_1^{\textrm{high}},\varvec{y}_2^{\textrm{high}},\ldots ,\varvec{y}_{P_{\textrm{DL}}}^{\textrm{high}} \right\}$ where $P_{\textrm{DL}}$ is the total number of patch images used for dictionary learning. We simultaneously optimize a the high-resolution dictionary $\varvec{D}^{\textrm{high}}$ and a matrix with sparse vectors $\varvec{X}$ as follows:

4 $$\begin{aligned} \left( \varvec{D}_{\textrm{est}}^{\textrm{high}},\varvec{X}_{\textrm{est}} \right) = \arg \min _{\left( \varvec{D}^{\textrm{high}}, \varvec{X} \right) } \Vert \varvec{Y}^{\textrm{high}} - \varvec{D}^{\textrm{high}} \varvec{X} \Vert _2^2 + \lambda \Vert \varvec{X} \Vert _1 \end{aligned}$$

where the first term represents the discrepancies between the high-resolution patch images $\varvec{Y}^{\textrm{high}}$ and the corresponding reconstructed images $\varvec{D}^{\textrm{high}} \varvec{X}$, and the second term is an $L_1$ regularization term for sparsity condition^36,37^. $\lambda$ is a regularization parameter that controls the sparsity. Note that the dictionaries are assumed to have arbitrary frequency elements in the proposed method by Eq. (4), whereas only specific high-frequency elements are considered for the reconstruction in pre-existing methods^10,26^. This generalized framework in the proposed method is formulated since various frequency elements should be considered for understanding rock textures, which include both low- and high-frequency elements, whereas specific high-frequency elements are rather important for efficiently obtaining face and object images with clear edges in computer graphics. The low-resolution dictionary $\varvec{D}_{\textrm{est}}^{\textrm{low}}$ is derived from the obtained high-resolution dictionary $\varvec{D}_{\textrm{est}}^{\textrm{high}}$ by using the downsampling matrix $\varvec{L}$ as follows: $\varvec{D}_{\textrm{est}}^{\textrm{low}}=\varvec{L} \varvec{D}_{\textrm{est}}^{\textrm{high}}$.

In the super-resolution step, a sparse vector is estimated that can reconstruct the low-resolution patch images $\tilde{\varvec{Y}}^{\textrm{low}}=\left\{ \tilde{\varvec{y}}_1^{\textrm{low}},\tilde{\varvec{y}}_2^{\textrm{low}},\ldots ,\tilde{\varvec{y}}_{P_{\textrm{SR}}}^{\textrm{low}} \right\}$ ($P_{\textrm{SR}}$: the total number of patch images for super-resolution) in terms of a small number of basis images. For appropriate reconstruction, a matrix with sparse vectors, $\tilde{\varvec{X}}$, is optimized by minimizing the following expression:

5 $$\begin{aligned} \tilde{\varvec{X}}_{\textrm{est}} = \arg \min _{\tilde{\varvec{X}}} \Vert \varvec{{\tilde{Y}}}^{\textrm{low}}- \varvec{D}_{\textrm{est}}^{\textrm{low}} \tilde{\varvec{X}} \Vert _2^2 +\lambda \Vert \tilde{\varvec{X}} \Vert _1 \end{aligned}$$

By assuming that high- and low-resolution images have the weight matrix $\tilde{\varvec{X}}$ in common, high-resolution patch images $\tilde{\varvec{Y}}_{\textrm{est}}^{\textrm{high}}$ can be reconstructed from the high-resolution dictionary and estimated weight matrix $\tilde{\varvec{X}}_{\textrm{est}}$ as follows:

6 $$\begin{aligned} \tilde{\varvec{Y}}_{\textrm{est}}^{\textrm{high}}= \varvec{D}_{\textrm{est}}^{\textrm{high}} \tilde{\varvec{X}}_{\textrm{est}}. \end{aligned}$$

The high-resolution reconstructed image is further refined by considering reconstruction in both the high- and low-resolution domains. See the Supplementary Material for further details.

### Settings for dictionary learning and super-resolution

The dictionary learning involves first creating a high-resolution dictionary. For this study, $P_{\textrm{DL}}=4964$ patches were prepared from six rock CT images used to conduct the dictionary creation. Individual CT images were $1856 \times 1856$ pixels.

**Figure 7:**
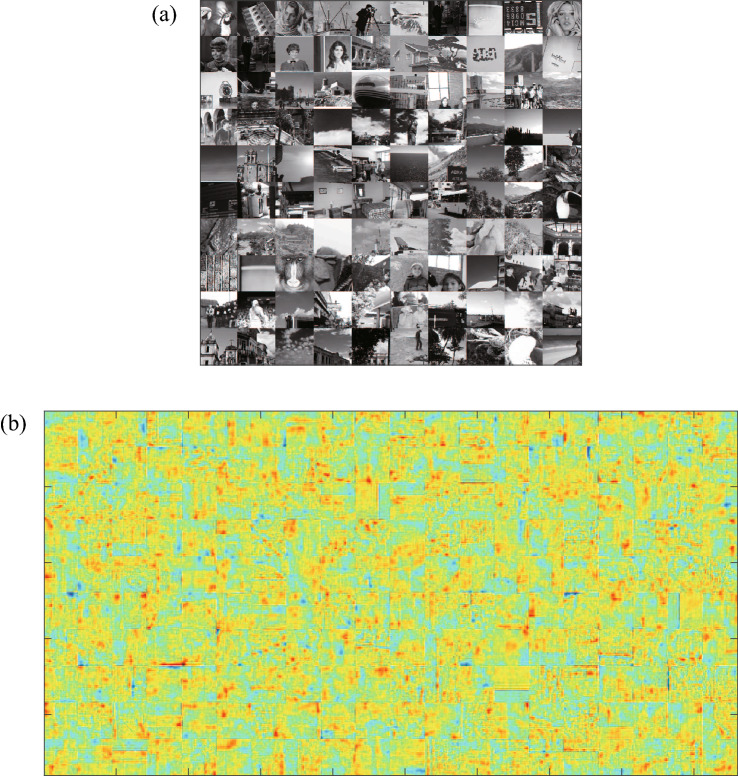
Dictionary trained by using the standard image datasets^41,42^: (**a**) images included in the standard image dataset and (**b**) basis images trained by using the standard image datasets.

**Figure 8:**
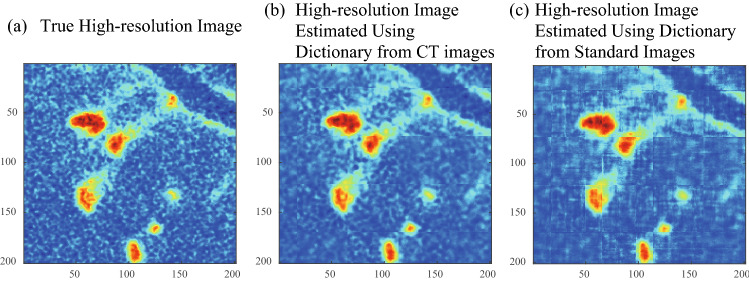
Comparison of high-resolution CT images estimated by using different dictionaries. (**a**) True high-resolution image , (**b**) high-resolution images estimated using dictionaries based on CT images (PSNR: 30.12, SSIM: 0.72), (**c**): high-resolution images estimated using dictionaries based on standard images (PSNR: 27.32, SSIM: 0.57). Voxel size of the high-resolution image was 10 $\upmu \hbox {m}$.

**Figure 9:**
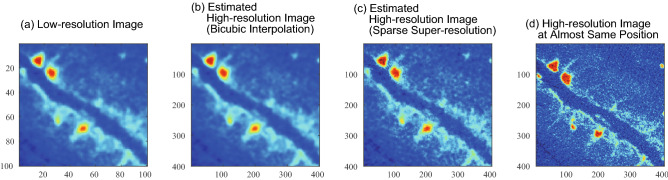
Application of the proposed method to directly obtained low-resolution image. (**a**) Low-resolution image directly obtained by X-ray CT scanner. (**b**) Estimated high-resolution image by bicubic interpolation. (**c**) Estimated high-resolution image by sparse super-resolution. (**d**) High-resolution image recorded at almost same position by X-ray CT scanner. Voxel size of the high-resolution image was 10 $\upmu \hbox {m}$.

The rock CT image used for training was divided into patches, and the average of the CT values for each patch was calculated. The data obtained by subtracting the average value from the CT value for each patch was used for training. The patch size of the high-resolution images was set to $48 \times 48$ pixels after some trial and error, and the number of basis images in the dictionary was set to $D=200$. When the initial high-resolution dictionary $\varvec{D}^{\textrm{high}}$ was fixed, the sparsity coefficients $\varvec{X}$ for the linear combination of high-resolution CT images were estimated. Then, when the estimated sparsity coefficients $\varvec{X}$ were fixed, a high-resolution dictionary $\varvec{D}^{\textrm{high}}$ suitable for sparse representation was obtained by imposing constraints to normalize the scales of the bases. We iterated these two tasks to obtain a high-resolution dictionary $\varvec{D}^{\textrm{high}}$ by using the result when the values converged. A low-resolution dictionary $\varvec{D}^{\textrm{low}}$ was created by downsampling the high-resolution dictionary by a factor of 1/4 using a smoothing filter.

The hyperparameters ($\lambda$, *c* and $\beta$) for dictionary learning were optimized by setting them to different values and selecting those that resulted in a small reconstruction error.

## Results and discussion

### Estimation from downsampled low-resolution images

The sparse super-resolution method was applied to estimating high-resolution rock CT images from low-resolution rock CT images (Fig. 1). Figure 3 shows an example of the estimation results: (a) the low-resolution CT images artificially prepared by downsampling the high-resolution CT image, (b) the estimation with bicubic interpolation, (c) estimation with the proposed sparse super-resolution, and (d) the true high-resolution image. First, the fine structures around localized red area is considered, which correspond to spinel grains. With bicubic interpolation, the boundary between red and yellow areas around localized red area was smoothly curved, and the intricate structures around the boundary were lost. In contrast, the proposed sparse super-resolution was able to reconstruct the complex structures around the boundary between the red (spinel grain) and yellow parts (magnetite rims replacing spinel).

Next, we focus on the linear structure in the upper right area of the images in Fig. 3, which corresponds to the serpentine veins. With bicubic interpolation, the boundary between the green and blue areas is overly smooth with a smooth contour. With the proposed sparse super-resolution, the intricate parts of the green and blue areas were reconstructed with a complex structure, which is similar to the true image. For example, some tube-like structures perpendicular to the serpentine veins can be observed in both the true image and image estimated by sparse super-resolution, while such tube-like structures were not reconstructed by bicubic interpolation. Therefore, the proposed sparse super-resolution accurately reconstructed the detailed structures of the serpentine vein and the reaction zone at its boundary.

Finally, the texture in the lower right area the images in Fig. 3 is considered, which corresponds to the mesh structure. The true image shows a complex texture with light blue pixels in a deep blue area. The sparse super-resolution reconstructed such complex structures. In contrast, bicubic interpolation reconstructed smooth textures in a deep blue area. These results show that the proposed sparse super-resolution framework reconstructed textures more accurately than the conventional bicubic interpolation, including spinel grains, their replacement textures, and serpentine veins.

To evaluate the effectiveness of the proposed framework for rock CT images in more detail, histograms of pixels in estimated high-resolution images are shown (middle subfigures of Fig. 3). These histograms may reflect some physical characteristics for a specific area in the rock CT images such as the modal abundances of minerals and porosities. Thus, it is important to evaluate histograms for the similarity between the true and estimated high-resolution images. The histograms for sparse super-resolution showed a smooth peak with pixel values between 1500 and 9000. The histogram for bicubic interpolation showed a sharp peak with pixel values between 2000 and 9000. The histogram for the true high-resolution image showed a smooth peak with pixel values between 1500 and 9000. These results suggest that the proposed sparse super-resolution method reconstructed the distribution of pixel values more precisely than bicubic interpolation.

The spatial distribution of pixel values is also evaluated as shown in the bottom row of Fig. 3. Here the spatial distribution of the pixel values is considered for the high-resolution images over a horizontal distance of $y=70{,}160$. Bicubic interpolation obtained a smoother spatial distribution than sparse super-resolution. When compared with the true high-resolution image, super-resolution accurately reproduced not only the global structure of the distribution (i.e., mineral grains and veins) but also fluctuations in the distribution (i.e., mineral replacement textures).

For a general validation of the proposed method, Fig. 4 shows the results at different position in the CT image. Fine structures in true high-resolution image, such as textures in the blue area and a rough boundary around the red area, were reconstructed by sparse super-resolution, whereas these structures were oversimplified by bicubic interpolation. This tendency can be confirmed in the histogram and pixel values in the cross section with the different methods. For example, the histogram obtained for the sparse super-resolution showed maximal counts, sharpness, and pixel value for maximal counts, which are more similar to the histogram of true high-resolution image than that obtained for the bicubic interpolation method (Fig. 4, middle). These results show that the basis images extracted from rock CT images play an important role in the estimation of complex structure.

### Characteristics of dictionary

For dictionary learning, the basis images were initially set randomly. As the dictionary learning proceeded, the spatial features hidden in the training CT image dataset were extracted, as shown in Fig. 5a. Note that the analyses for the basis images can be conducted since the sparse super-resolution is a white-box type method, which is in cotrast to black-box type methods, such as deep learning-based super-resolution methods^27–29^. All basis images showed not only with large-scale (i.e., low spatial frequency) structures but also small-scale (i.e., high spatial frequency) structures. The coexistence of structures at different scales required enhanced estimation accuracy. We evaluated the estimation accuracy of the proposed method in terms of the peak signal-to-noise ratio (PSNR) and the structural similarity index measure (SSIM)^9,38,39^, as shown in Fig. 6. See the Supplementary Material for the definitions of these indices. The PSNR increased with the iterations of dictionary learning but was quite low when the initial randomly set basis images were used. The SSIM also increased with the number of iterations. The improvement in image quality according to these indices corresponds to the adaptation of basis images included in the dictionary.

The basis images were further analyzed by using clustering and dimension reduction techniques. For clustering, the *k*-means$++$ algorithm was applied to vectors representing basis images^40^. This algorithm avoids the initial-value dependence of the original *k*-means clustering. To visualize the results in two-dimensional space, a dimension reduction method called the *t*-distributed stochastic neighbor embedding (*t*-SNE) method^40^ was applied to the clustering results. As shown in Fig. 5b,c, the basis vectors were separated into eight clusters. The basis images had localized red areas around specific regions and complex textures. The level of localization depended on the cluster. Red areas in cluster 6 were less localized but more dispersed, while the red areas in clusters 1, 2, and 4 were more localized. Cluster 1 had a localized red area (i.e., high CT number) toward the top, whereas cluster 2 had a similar area but toward the bottom.

To investigate the effect of source images on dictionary learning and super-resolution, we conducted dictionary learning using other images included in the standard image datasets called IAPR TC-12 (International Association of Pattern Recognition, Technical Committee 12) collection^41^ and SIDBA (Standard Image Data-BAse)^42^, including people, buildings, and landscapes (Fig. 7a). The dictionary obtained from standard image dataset (Fig. 7b) includes clearer boundaries and more straight-line structure than the dictionary obtained from the rock CT images (Fig. 5a). A comparison between the high-resolution images obtained from the two dictionaries (Fig. 8) indicates that the dictionary obtained from the rock CT images (Fig. 8b) provided more accurate high-resolution images than the dictionary obtained from the standard images (Fig. 8c). The high-resolution image reconstructed using the dictionary from rock CT images reproduced fine structures including textures and the boundary around red regions, while the high-resolution image reconstructed using the dictionary from standard images included many sharp structures not seen in the true high-resolution image. The quantitative evaluation indices also demonstrated the superiority of the dictionary obtained from rock CT images, which had a greater PSNR (30.12) than the dictionary obtained from standard images (27.32) as well as SSIM. These results suggest that the basis images extracted by sparse super-resolution include the essential characteristics of the rock textures.

### Application to recorded low-resolution images

Here, the proposed method was applied to low-resolution images, which were directly recorded by the CT scanner. The high-resolution images (Fig. 9b,c) were estimated from a low-resolution image (Fig. 9a) directly recorded by the CT scanner. The high-resolution image that was estimated by bicubic interpolation (Fig. 9b) shows a rather simple and clear structure. In contrast, the high-resolution image that was estimated by sparse super-resolution (Fig. 9c) successfully shows more complex structures. For example, in Fig. 9c, the detailed structures of spinel are shown by red areas in the upper-left region, and complex mesh textures are shown by deep blue areas.

The high-resolution image estimated from directly recorded low-resolution image by the sparse super-resolution (Fig. 9c) is found to be more similar to the high-resolution image recorded at almost same position directly (Fig. 9d), compared with that estimated by bicubic interpolation (Fig. 9b). Note that the position of the high-resolution image (Fig. 9d) is almost the same as that of the low-resolution image but is not the same exactly. From these results, the proposed sparse super-resolution is found to be effective for estimating high-resolution rock CT image.

Note that there is still a discrepancy between the high-resolution image that was estimated from the recorded low-resolution image by the sparse super-resolution (Fig. 9c) and the high-resolution image that was recorded at almost the same position (Fig. 9d). This is probably due to the down-sampling matrix simply that was assumed in the present study. A new dictionary learning method for estimating the accurate relationship between high- and low-resolution images with appropriate position correspondence is required to reduce this discrepancy and realize more accurate estimation in future studies.

## Concluding remarks

In this study, we have proposed a method to apply sparse super-resolution to rock CT images. In contrast to interpolation algorithms, the proposed method estimates the image by sparse representation and dictionary learning to reconstruct missing information in the low-resolution image. The experimental results showed that sparse super-resolution method was better at reconstructing details of rock CT images than bicubic interpolation. The superiority of the proposed method was quantified by PSNR and SSIM. These results confirmed that the proposed method extracts the important features of rock CT images and obtains better results than conventional interpolation, which may be a significant contributions to practical applications.

The medical CT scanner used for the long geological core analyses provides low resolution images ($>0.1$ voxel size) on the mineral-scale microstructures but is strong tool for quantitative analyses of the continuous geological structures over 100 m. In contrast, the researchers carry out in the detailed microstructural analyses from the limited samples in their labs, by using for example high-resolution X-ray CT. Therefore, if we develop a super-resolution techniques to link the lab micro CT scanner and medical CT in D/Y *Chikyu*, we can connect the phenomena from nano to micro scale to kilometer scales. This study only shows the super-resolution in the same scanner with different magnification or artificial images created by simple down sampling, it is important to find the way the realistic down sampling that enables to link over the images taken by the different scanners.

## Supplementary Information

Supplementary Information.

## Data Availability

The original and treated X-ray CT images are available from 10.6084/m9.figshare.5458696. Codes and further data can be made available by the corresponding author (T.O.) upon reasonable request.
