# Supplementary material for: Super-resolution of X-ray CT images of rock samples by sparse representation: applications to the complex texture of serpentinite
Source: Sci Rep. 2023 Apr 24;13:6648. doi: 10.1038/s41598-023-33503-6 (PMC10126017; doi:10.1038/s41598-023-33503-6)
Supplement: Supplementary file 1 — Supplementary Information. [file 41598_2023_33503_MOESM1_ESM.pdf]

Supplementary Material: Super-resolution of X-ray CT Images of Rock Samples by Sparse Representation: Applications to the Complex Texture of Serpentinite

Toshiaki Omori[1,2,3*], Shoi Suzuki[1], Katsuyoshi Michibayashi[4], and Atsushi Okamoto[5]

[1]Department of Electrical and Electronic Engineering, Graduate School of Engineering, Kobe University, Kobe, 657-8501, Japan
[2]Center for Mathematical and Data Sciences, Kobe University, Kobe, 657-8501, Japan
[3]Center of Optical Scattering Image Science, Kobe University, Kobe, 657-8501, Japan
[4]Department of Earth and Planetary Sciences, Graduate School of Environmental Studies, Nagoya University, Nagoya, 464-8601, Japan
[5]Department of Environmental Studies for Advanced Society, Graduate School of Environmental Studies, Tohoku University, Sendai, 980-8579, Japan
[*]omori@eedept.kobe-u.ac.jp

Detailed formulation of sparse super-resolution for rock CT images

Here, we describe the detailed formulation of the proposed sparse super-resolution for rock CT images. A small area of images (i.e., a patch) $\boldsymbol{y}_i$ is assumed to be expressed by basis images with a sparse vector. Corresponding high- and low-resolution images are assumed to have a common sparse vector as follows:

$$\boldsymbol{y}_i^{\text{high}} = x_{i,1}\boldsymbol{d}_1^{\text{high}} + x_{i,2}\boldsymbol{d}_2^{\text{high}} + \cdots + x_{i,D}\boldsymbol{d}_D^{\text{high}} \tag{S1}$$

$$\boldsymbol{y}_i^{\text{low}} = x_{i,1}\boldsymbol{d}_1^{\text{low}} + x_{i,2}\boldsymbol{d}_2^{\text{low}} + \cdots + x_{i,D}\boldsymbol{d}_D^{\text{low}} \tag{S2}$$

where dictionaries for high-resolution and low resolution images are expressed by $\left\{\boldsymbol{d}_1^{\text{high}}, \boldsymbol{d}_2^{\text{high}}, \cdots, \boldsymbol{d}_D^{\text{high}}\right\}$ and $\left\{\boldsymbol{d}_1^{\text{low}}, \boldsymbol{d}_2^{\text{low}}, \cdots, \boldsymbol{d}_D^{\text{low}}\right\}$, respectively.

The sparse super-resolution comprises two steps: dictionary learning using high-resolution images, and reconstruction of the high-resolution image by applying sparse coefficients obtained from the low-resolution image to high-resolution image representation (Fig. 1 in main article). In both steps, the L_1 norm is used to realize sparse representation. The size of the image patches is adjusted by try and error.

In the dictionary learning step, we obtain a dictionary with basis images $\boldsymbol{D}^{\text{high}} = \left\{\boldsymbol{d}_1^{\text{high}}, \boldsymbol{d}_2^{\text{high}}, \cdots, \boldsymbol{d}_D^{\text{high}}\right\}$ (D: the number of basis images) from a set of patch images $\boldsymbol{Y}^{\text{high}} = \left\{\boldsymbol{y}_1^{\text{high}}, \boldsymbol{y}_2^{\text{high}}, \cdots, \boldsymbol{y}_{P_{\text{DL}}}^{\text{high}}\right\}$ (P_{DL}: the total number of patch images used for dictionary learning, which are obtained from high-resolution images). Here, we simultaneously optimize a high-resolution dictionary $\boldsymbol{D}^{\text{high}}$ and a matrix with sparse vectors $\boldsymbol{X}$ as follows:

$$\left(\boldsymbol{D}_{\text{est}}^{\text{high}}, \boldsymbol{X}_{\text{est}}\right) = \arg \min_{\left(\boldsymbol{D}^{\text{high}}, \boldsymbol{X}\right)} \|\boldsymbol{Y}^{\text{high}} - \boldsymbol{D}^{\text{high}}\boldsymbol{X}\|_2^2 + \lambda\|\boldsymbol{X}\|_1 \tag{S3}$$

where the first term represents the discrepancies between high-resolution patch images $\boldsymbol{Y}^{\text{high}}$ and the corresponding reconstructed images $\boldsymbol{D}^{\text{high}}\boldsymbol{X}$, and the second term is an L_1 regularization term for the sparsity condition. Here, λ is a regularization parameter that controls the sparsity. Optimization is performed iteratively in Eq. (S3) for one of two factors $\left(\boldsymbol{D}^{\text{high}}, \boldsymbol{X}\right)$ as follows:

$$\boldsymbol{X}_* = \arg\min_{\boldsymbol{X}} \|\boldsymbol{Y}^{\text{high}} - \boldsymbol{D}_*^{\text{high}}\boldsymbol{X}\|_2^2 + \lambda\|\boldsymbol{X}\|_1 \tag{S4}$$

$$\boldsymbol{D}_*^{\text{high}} = \arg\min_{\boldsymbol{D}^{\text{high}}} \|\boldsymbol{Y}^{\text{high}} - \boldsymbol{D}^{\text{high}}\boldsymbol{X}_*\|_2^2, \quad \|\boldsymbol{d}_i^{\text{high}}\|_2^2 \le 1 \quad (i = 1, 2, \cdots, D) \tag{S5}$$

where $\boldsymbol{X}_*$ and $\boldsymbol{D}_*^{\mathrm{high}}$ are the tentatively estimated sparse vector and dictionary, respectively. Note that the second term in Eq. (S4) is a constraint for sparsity, whereas the inequality in Eq. (S5) removes the scaling ambiguity. Minimizations in Eqs. (S4) and (S5) are conducted by the least absolute shrinkage and selection operator method and quadratically constrained quadratic problem, respectively. The low-resolution dictionary $\boldsymbol{D}_{\mathrm{est}}^{\mathrm{low}}$ is derived from the obtained high-resolution dictionary $\boldsymbol{D}_{\mathrm{est}}^{\mathrm{high}}$ by using a downsampling matrix $\boldsymbol{L}$ as $\boldsymbol{D}_{\mathrm{est}}^{\mathrm{low}} = \boldsymbol{L}\boldsymbol{D}_{\mathrm{est}}^{\mathrm{high}}$.

In the super-resolution step, we first estimate a sparse vector that can reconstruct low-resolution patch images $\tilde{\boldsymbol{Y}}^{\mathrm{low}} = \left\{\tilde{\boldsymbol{y}}_1^{\mathrm{low}}, \tilde{\boldsymbol{y}}_2^{\mathrm{low}}, \cdots, \tilde{\boldsymbol{y}}_{P_{\mathrm{SR}}}^{\mathrm{low}}\right\}$ (P_{SR}: the total number of patch images for super-resolution) in terms of a small number of basis images. For appropriate reconstruction, a matrix with sparse vectors, $\tilde{\boldsymbol{X}}$, is optimized by minimizing the following expression:

$$\tilde{\boldsymbol{X}}_{\mathrm{est}} = \arg\min_{\tilde{\boldsymbol{X}}} \|\tilde{\boldsymbol{Y}}^{\mathrm{low}} - \boldsymbol{D}_{\mathrm{est}}^{\mathrm{low}}\tilde{\boldsymbol{X}}\|_2^2 + \lambda\|\tilde{\boldsymbol{X}}\|_1 \tag{S6}$$

In each patch, the following optimization is individually conducted:

$$\tilde{\boldsymbol{x}}_{i,est} = \arg\min_{\tilde{\boldsymbol{x}}} \|\tilde{\boldsymbol{y}}_i^{\mathrm{low}} - \boldsymbol{D}_{\mathrm{est}}^{\mathrm{low}}\tilde{\boldsymbol{x}}\|_2^2 + \lambda\|\tilde{\boldsymbol{x}}\|_1 \tag{S7}$$

Basically, the high-resolution patch image $\tilde{\boldsymbol{y}}_{i,\mathrm{est}}$ can be reconstructed by using the high-resolution dictionary $\boldsymbol{D}_{\mathrm{est}}^{\mathrm{high}}$ and the estimated sparse vector $\tilde{\boldsymbol{x}}_{i,\mathrm{est}}$ as follows:

$$\tilde{\boldsymbol{y}}_{i,\mathrm{est}} = \boldsymbol{D}_{\mathrm{est}}^{\mathrm{high}}\tilde{\boldsymbol{x}}_{i,\mathrm{est}} \tag{S8}$$

However, the above individual optimization may induce unnatural boundaries between neighboring patches. To avoid this effect, we considered optimization with an overlap between the current target patch and previously reconstructed patches as follows:

$$\tilde{\boldsymbol{x}}_{i,\mathrm{est}} = \arg\min_{\tilde{\boldsymbol{x}}} \|\tilde{\boldsymbol{y}}_i^{\mathrm{low}} - \boldsymbol{D}_{\mathrm{est}}^{\mathrm{low}}\tilde{\boldsymbol{x}}\|_2^2 + \beta\sum_{j\in N(i)} \|\tilde{\boldsymbol{y}}_j^{\mathrm{high}} - \boldsymbol{P}_{i,j}\boldsymbol{D}_{\mathrm{est}}^{\mathrm{high}}\tilde{\boldsymbol{x}}\|_2^2 + \lambda\|\tilde{\boldsymbol{x}}\|_1 \tag{S9}$$

where $\boldsymbol{P}_{i,j}$ extracts the region of overlap between the current target patch i and a previously reconstructed high-resolution patch image. Here, $N(i)$ is a set of patches neighboring the current patch i whose high-resolution patch images have already been reconstructed. β is a positive constant.

By assuming that a weight matrix $\tilde{\boldsymbol{X}}$ is common between high-resolution and low-resolution images, high-resolution patch images $\tilde{\boldsymbol{Y}}_0^{\mathrm{high}}$ can be reconstructed from the high-resolution dictionary and the estimated weight matrix $\tilde{\boldsymbol{X}}_{\mathrm{est}}$ as follows:

$$\tilde{\boldsymbol{Y}}_0^{\mathrm{high}} = \boldsymbol{D}_{\mathrm{est}}^{\mathrm{high}}\tilde{\boldsymbol{X}}_{\mathrm{est}}. \tag{S10}$$

The high resolution patch images $\tilde{\boldsymbol{Y}}_0^{\mathrm{high}}$ may not satisfy the constraint between high-resolution and low-resolution images $\boldsymbol{Y}^{\mathrm{low}} = \boldsymbol{L}\boldsymbol{Y}^{\mathrm{high}}$. To satisfy the constraint, we consider the following update for high-resolution images:

$$\tilde{\boldsymbol{Y}}_*^{\mathrm{high}} = \arg\min_{\tilde{\boldsymbol{Y}}^{\mathrm{high}}} \|\boldsymbol{Y}^{\mathrm{low}} - \boldsymbol{L}\tilde{\boldsymbol{Y}}^{\mathrm{high}}\|_2^2 + c\|\tilde{\boldsymbol{Y}}^{\mathrm{high}} - \tilde{\boldsymbol{Y}}_0^{\mathrm{high}}\|_2^2 \tag{S11}$$

where c is a positive constant. The first and second terms in Eq. (S11) respectively correspond to the constraint on the relation between high and low resolution images for reconstruction and the constraint on reconsructed high-resolution image by means of sparse modeling. Eq. (S11) is optimized by the steepest decent. The iterative equation for the high-resolution image is expressed as follows:

$$\tilde{\boldsymbol{Y}}_{k+1}^{\mathrm{high}} = \tilde{\boldsymbol{Y}}_k^{\mathrm{high}} + \gamma\left\{\boldsymbol{L}^T\left(\boldsymbol{Y}^{\mathrm{low}} - \boldsymbol{L}\tilde{\boldsymbol{Y}}_k^{\mathrm{high}}\right) + c\left(\tilde{\boldsymbol{Y}}_k^{\mathrm{high}} - \tilde{\boldsymbol{Y}}_0^{\mathrm{high}}\right)\right\} \tag{S12}$$

where $\tilde{\boldsymbol{Y}}_k^{\mathrm{high}}$ is the obtained high-resolution image at iteration k. Here γ is a positive constant. We regard $\tilde{\boldsymbol{Y}}_k^{\mathrm{high}}$ at sufficiently large k as estimate high-resolution image $\tilde{\boldsymbol{Y}}_*^{\mathrm{high}}$.

Image quality indices

We considered two kinds of image quality indices for comparing two images $I = \{i_{x,y}\}$ and $J = \{j_{x,y}\}$ (x, y: pixel number in two-dimensional space). If one of the two images is the true high-resolution image and the other is the estimated image, the following indices can be used to quantify the performance of super-resolution method.

The signal-to-peak noise ratio (PSNR) is defined by using the mean-squared error:

$$\mathrm{PSNR} = 20\log_{10}\frac{\mathrm{MAX}}{\sqrt{\mathrm{MSE}}} \tag{S13}$$

where MSE is the mean-squared error between images I and J as follows:

$$\mathrm{MSE} = \frac{1}{n}\sum_{x,y}(i_{x,y} - j_{x,y})^2 \tag{S14}$$

Here, n is the total number of pixels. MAX expresses the maximum pixel value that an image format can take. As expressed in Eq. (S14), the PSNR uses discrepancy between images in the pixel level.

The structural similarity index measure (SSIM) is defined by the average and variance of each image as follows:

$$\mathrm{SSIM} = \frac{(2\mu_i\mu_j + c_1)(2\sigma_{ij} + c_2)}{(\mu_i^2 + \mu_j^2 + c_1)(\sigma_i^2 + \sigma_j^2 + c_2)} \tag{S15}$$

where μ_x and μ_y are the averaged pixel values of images I and J, respectively. σ_i and σ_j are the variances of images I and J, respectively, while σ_{ij} is the covariance of images I and J. Here, c_1 and c_2 are constants. As expressed in Eq. (S15), the SSIM evaluates the discrepancy between images using statistics for the image structure rather than discrepancy between images in the pixel level.
